# Mind the gap: imaging spectrum of abdominal ventral hernia repair complications

**DOI:** 10.1186/s13244-019-0730-x

**Published:** 2019-03-29

**Authors:** Aruna R. Patil, Shrivalli Nandikoor, Himansu Shekar Mohanty, Satyajit Godhi, Ravishankar Bhat

**Affiliations:** 1 Department of Radiology, Apollo Hospitals, opp IIM, Bangalore, Karnataka 560078 India; 2 Surgical Gastroenterology, Apollo Hospitals, opp IIM, Bangalore, Karnataka 560078 India

**Keywords:** Hernia, Mesh, Computed tomography, Magnetic resonance imaging, Ultrasonography

## Abstract

Ventral hernia repair with or without mesh placement is a commonly done procedure. Laparoscopic approach is more preferred than open in recent surgical practice. Complications occur as like any other abdominal surgeries and are dependent on multiple factors. Complications such as collections, adhesions, and related changes are non-specific. Specific complications related to hernia repair include recurrent hernia, mesh infection, mesh migration, and fistula formation. Post inguinal hernia repair chronic inguinal pain is gaining more attention with increasing use of image-guided nerve interventions for symptomatic management. Imaging plays a vital role in defining and delineating the type and extent of complications. Prior knowledge of the surgical indication and technique helps in better imaging interpretation of complications. This article describes the role of imaging in diagnosis of complications in general ventral hernia surgery setting.

## Key messages

1. Multiple factors (type of hernia, surgical technique, and patient comorbidities) influence the complication rate and type in post ventral hernia repair.
2. Multimodality imaging (especially CT) is essential for the adequate diagnosis of various complications that can occur post hernia repair.
3. Combined surgeon–radiologist participation is imperative in diagnosis and prompt management.

## Introduction

Ventral hernia repair with or without mesh placement is a commonly done procedure either using laparoscopic approach or open techniques with former preferred than latter. Complications occur as like any other abdominal surgeries and are dependent on multiple factors. Complications such as collections, adhesions, and related changes are nonspecific and are common to any abdominal surgery. Specific complications related to hernia repair include recurrent hernia, mesh infection, mesh migration, fistula formation, and infertility. Post inguinal hernia repair chronic inguinal pain is being recognized more frequently with increasing use of image-guided nerve interventions for symptomatic management. Imaging plays a vital role in defining and delineating the type and extent of complications. Prior knowledge of the surgical indication and technique helps in better imaging interpretation of complications.

The various complications that can be encountered in post ventral hernia repair are summarized in Table 1. The overall incidence has a wide range [1]. There are a variety of factors that influence the occurrence of complications in a post hernia repair. Complication rate depends on the type of hernia that is repaired, the surgical technique used including the mesh type, and the patient factors. Factors that influence the complications are summarized in Table 2.

**Table 1:** Complications of hernia repair

Complications of hernia repair	
• Seroma	
• Hematoma	
• Adhesions and small bowel obstruction	
• Recurrent hernia	
• Mesh infection	
• Mesh migration	
• Fistulization with adjacent viscera	
• Post hernia repair chronic pain	
• Infertility

**Table 2:** Factors influencing complications

Factors influencing complications	
• Type and content of hernia	
• Surgical technique and mesh selection	
- Open vs laparoscopic	
- Instrument sterility	
- Mesh properties	
• Patient factors	

### Factors influencing complications

- Type of hernia: Higher chances of complications are seen with repair of parastomal hernia, recurrent hernia, and hernias with large and multiple defects (Swiss cheese pattern). The contents of the hernia also influence the complication rate with bowel relatively more predisposing than omentum or mesentery [2]. Hernias that require bowel anastomoses are relatively more prone as the surgical times are prolonged and its inherent predisposal due to bowel handling.
- Surgical technique and type of mesh: Onlay techniques show a higher complication rate than laparoscopic approach [3, 4]. Use of mesh in hernia repairs has reduced the complication rates compared to direct suturing. Even with mesh usage, inadequate fixation of the mesh, shrinkage of mesh can lead to recurrent hernia. Hence exact pre-operative assessment of the size and multiplicity of the defects is necessary for mesh selection. Mesh is a foreign body and various materials from metallic to biologic have been used. In the present surgical practice, the most commonly used mesh is polypropylene based with modifications in internal make, layering, compositeness, etc. and is quite variable with each center of practice and surgeon selection. The inherent characteristics of the mesh influence the outcome. Mesh material, pore size, weight, and filament type all are shown to alter the outcomes [5].

Instrument sterility is a prime requisite and an indispensable factor for surgical outcomes. In spite of strict sterilization techniques, this is still a prevailing problem in developing countries with increasing occurrence of tough and atypical microbes that resist routine sterilization techniques.

c. Patient factors: Defective or delayed healing and predisposition to infection is seen in patients with smoking, diabetes, obesity, and on steroid intake. In a study by Vidovic et al. [6], the hernia recurrence rate was 30.3%, with recurrence occurring more with tissue repair than with prosthesis. The authors stated that the recurrence was influenced by type of repair, obesity, hernia size, wound healing disorders, and some chronic comorbidities.

#### Imaging in complications

**Fig. 1:**
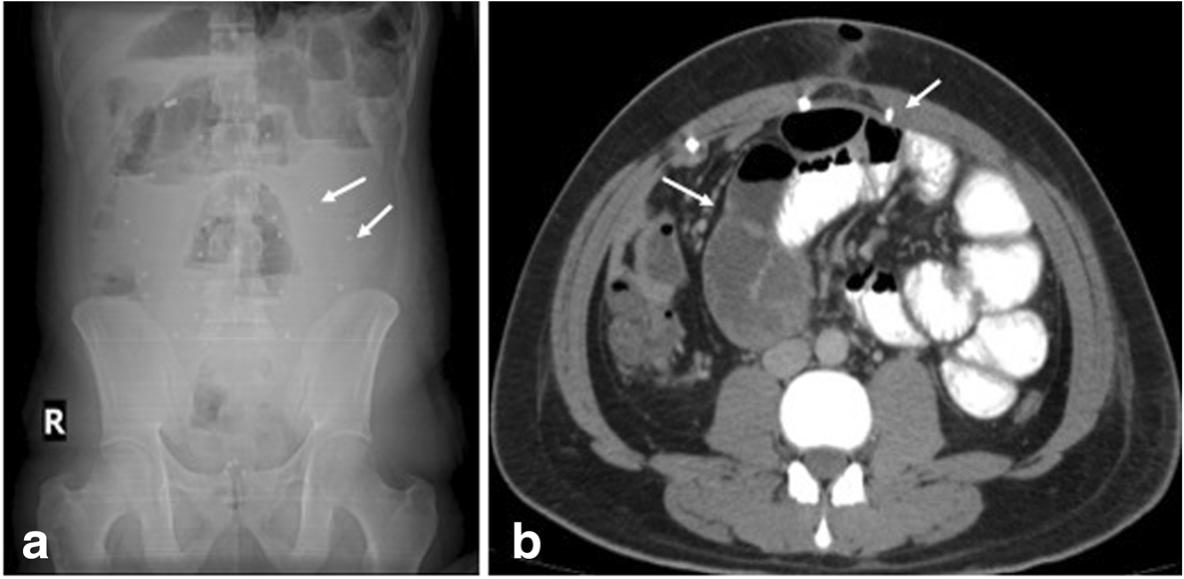
**a** Erect abdominal radiograph shows features of small bowel obstruction (multiple air fluid levels in dilated loops) with evidence of radiodense tackers (arrows) suggesting mesh placement. **b** CECT axial section of the same patient shows the radiodense tackers (short arrow) and adhesion as the cause of bowel obstruction (long arrow)

**Fig. 2:**
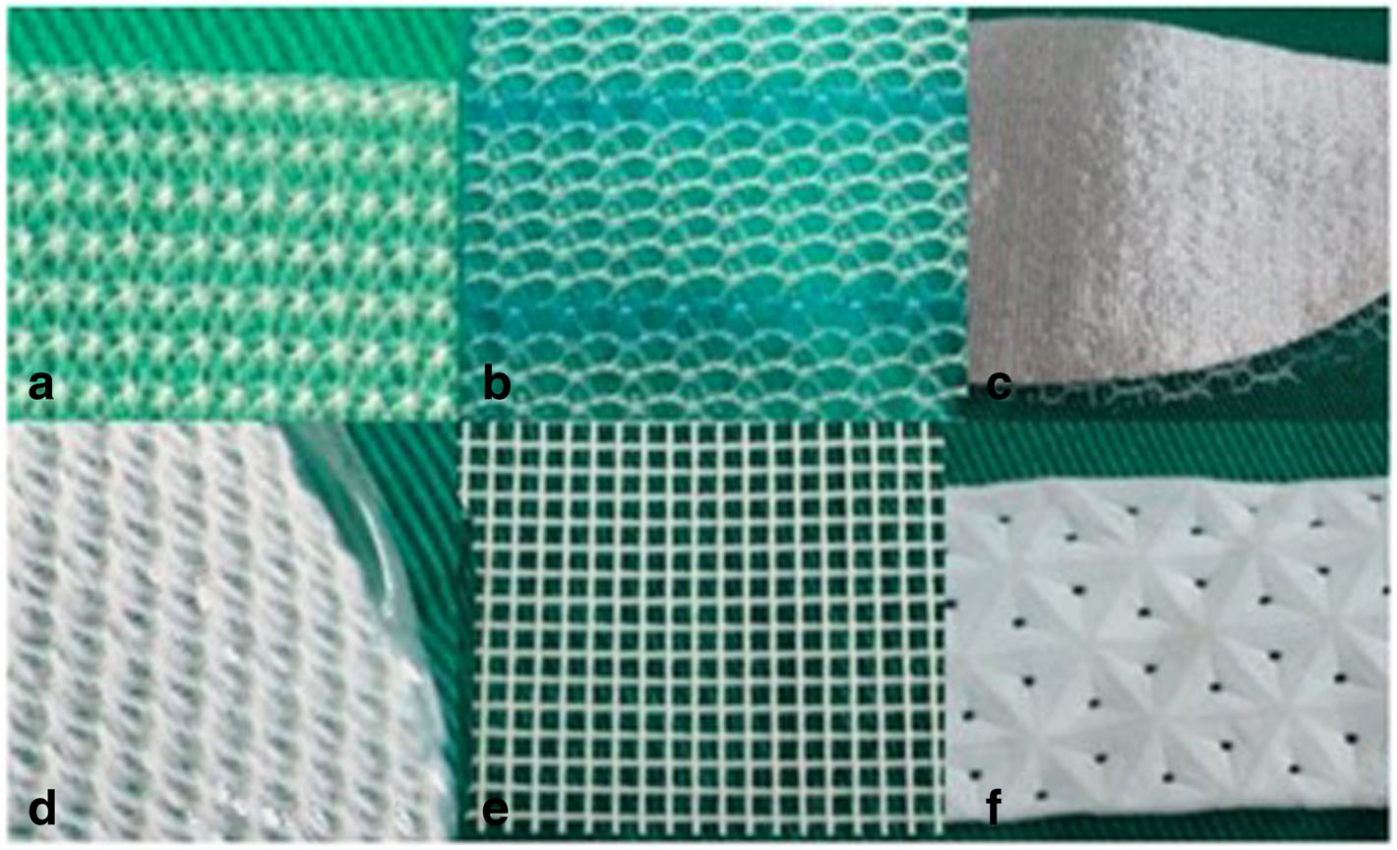
Different types of mesh. **a** Polypropylene. **b** Polypropylene polyglecaprone. **c** Polypropylene—polydioxanon. **d** Polyester/collagen. **e** Polyester. **f** Polytetrafluoroethylene (PTFE) (with permission from reference [6])

**Fig. 3:**
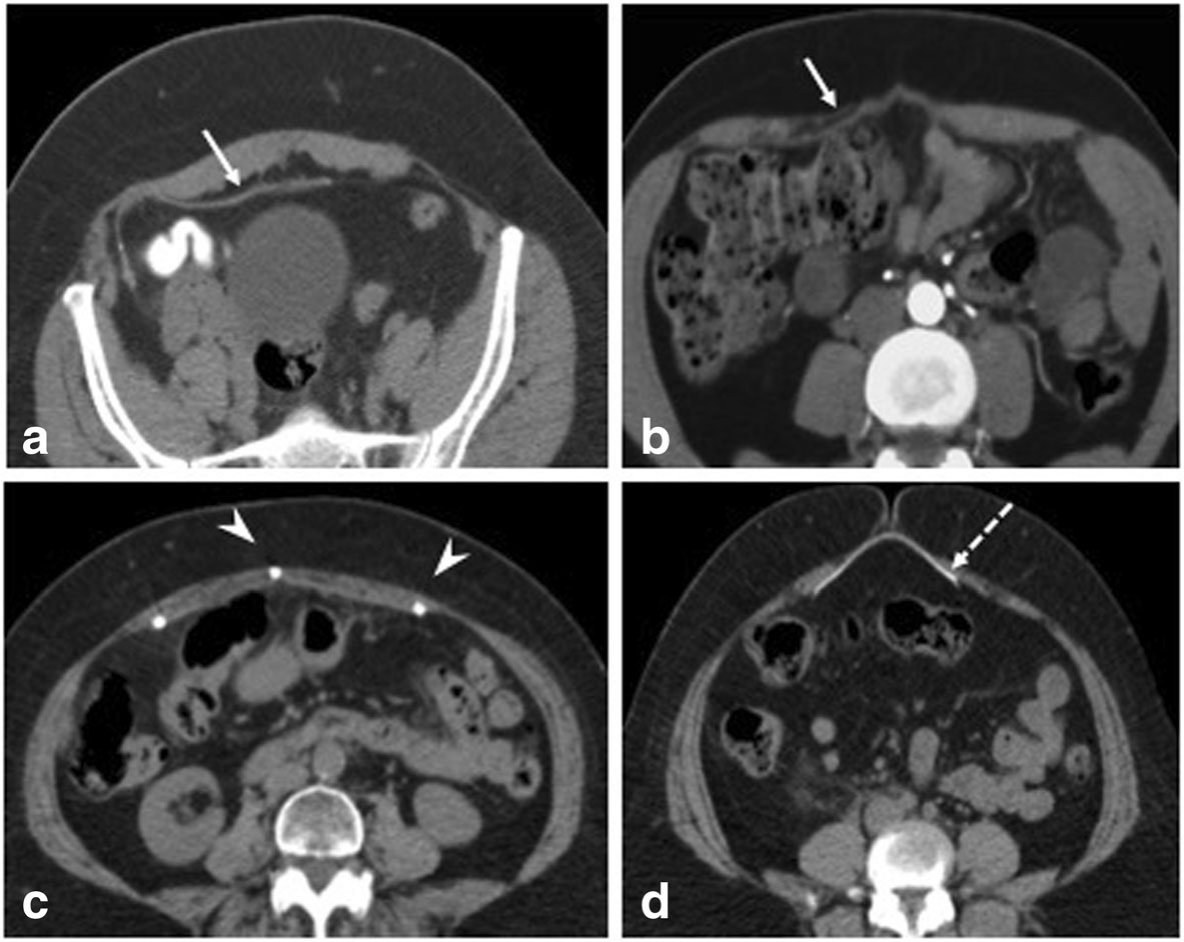
Mesh appearances on CT axial sections. **a** Preperitoneal placement of Prolene-based mesh appearing isodense to muscle. Presence of fat can aid in better visualization of the mesh. **b** Inlay Prolene-based mesh placement (arrow). **c** Laparoscopic intraperitoneal placement of mesh with tackers (arrowheads). **d** Mesh made of PTFE appearing radiodense (dashed arrow)

Awareness of the type of hernia repair and interaction with the surgeon is important for better understanding of the postoperative field and hence useful reporting contributions. Commonly used investigations are ultrasonography (USG) and CT [7]. Radiographs are used in suspected cases of small bowel obstruction or perforation (Fig. 1). CT aids in characterization of complications especially with use of contrast (oral -positive/ neutral, intravenous) that gives details about collections and bowel involvement. Various types of mesh are used in current practice with differences in composite material and structure (Fig. 2). Polypropelene or polyester mesh are preferred for extraperitoneal placement due to light weight and large pores hence lower infection risk. Mesh that is used for laparoscopic repair generally have a protective membrane or film to reduce adhesions with intraperitoneal structures [5, 8]. The major factors that determine mesh visibility on CT are density, structure (woven or knitted), and thickness. Polypropylene, polyester-based mesh is isodense to muscle and hence not visualized unless with a fat interface or metallic tackers. Polytetrafluoroethylene (PTFE) based are hyperdense and are readily seen (Fig. 3). Composite mesh made with different layers is visible on CT if composed of PTFE and again depends on the thickness used.

**Fig. 4:**
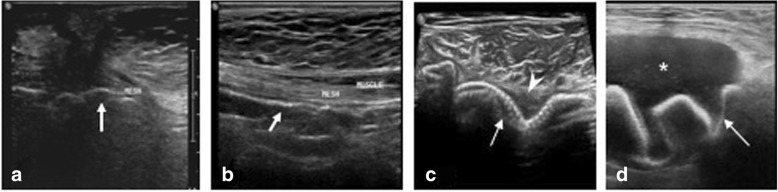
Mesh appearances on USG using high-resolution linear probe. **a** Wavy echogenic appearance of mesh (arrow) in a post umbilical hernia repair. **b** Inlay placement of mesh appearing echogenic on USG (arrow). **c** Early postoperative USG shows wavy echogenic mesh (arrow) made prominent by surrounding thin seroma (arrowhead). **d** Mesh migration (arrow) into a collection (*) in a post inguinal hernia repair

Ultrasound also shows variable appearance [9, 10] (Fig. 4) and presence of fluid around generally enhances its echogenicity and plays an important role in diagnosing mesh migration and infection. The study and modality selection hence must be tailored according to the clinical presentation with an ability to complement with an additional modality.

**Fig. 5:**
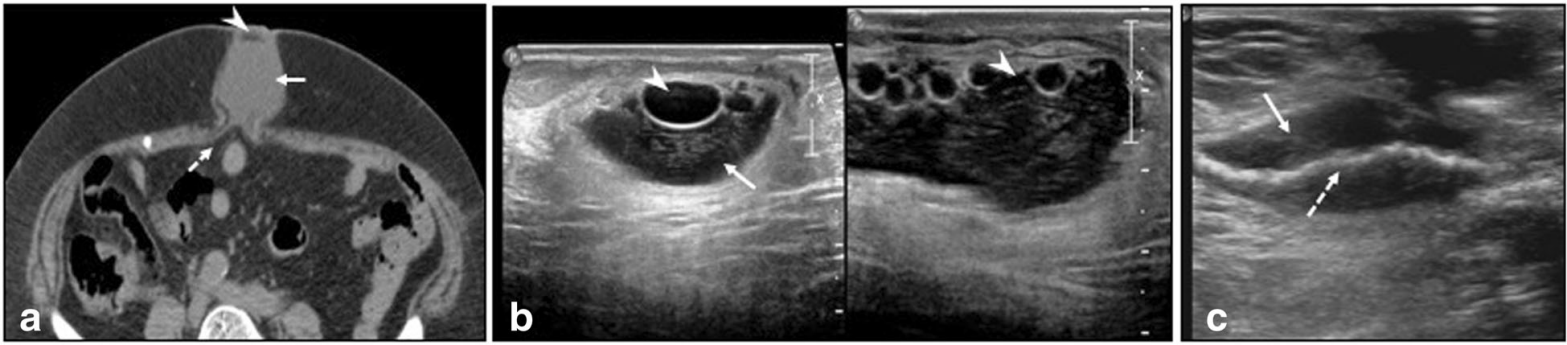
Varying appearances of seroma post hernia repair. **a** Plain CT in axial section shows seroma (solid arrow) with fat levels (arrowhead). Dashed arrow is the mesh. **b** USG shows floating fat locules (arrowheads) secondary to tissue dissection within the seroma (arrow). **c** USG shows seroma (solid arrow) around the mesh (dashed arrow) in an early postoperative hernia repair

**Fig. 6:**
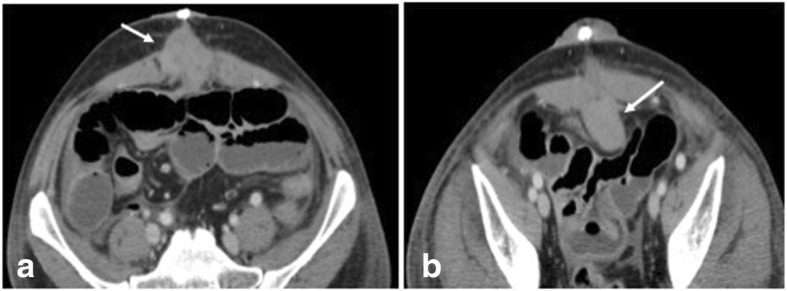
**a** CECT in axial section shows hyperdense parietal wall hematoma (arrow) in a patient with post open ventral hernia repair. **b** CECT axial section at inferior level shows preperitoneal extension of the hematoma (arrow)

**Fig. 7:**
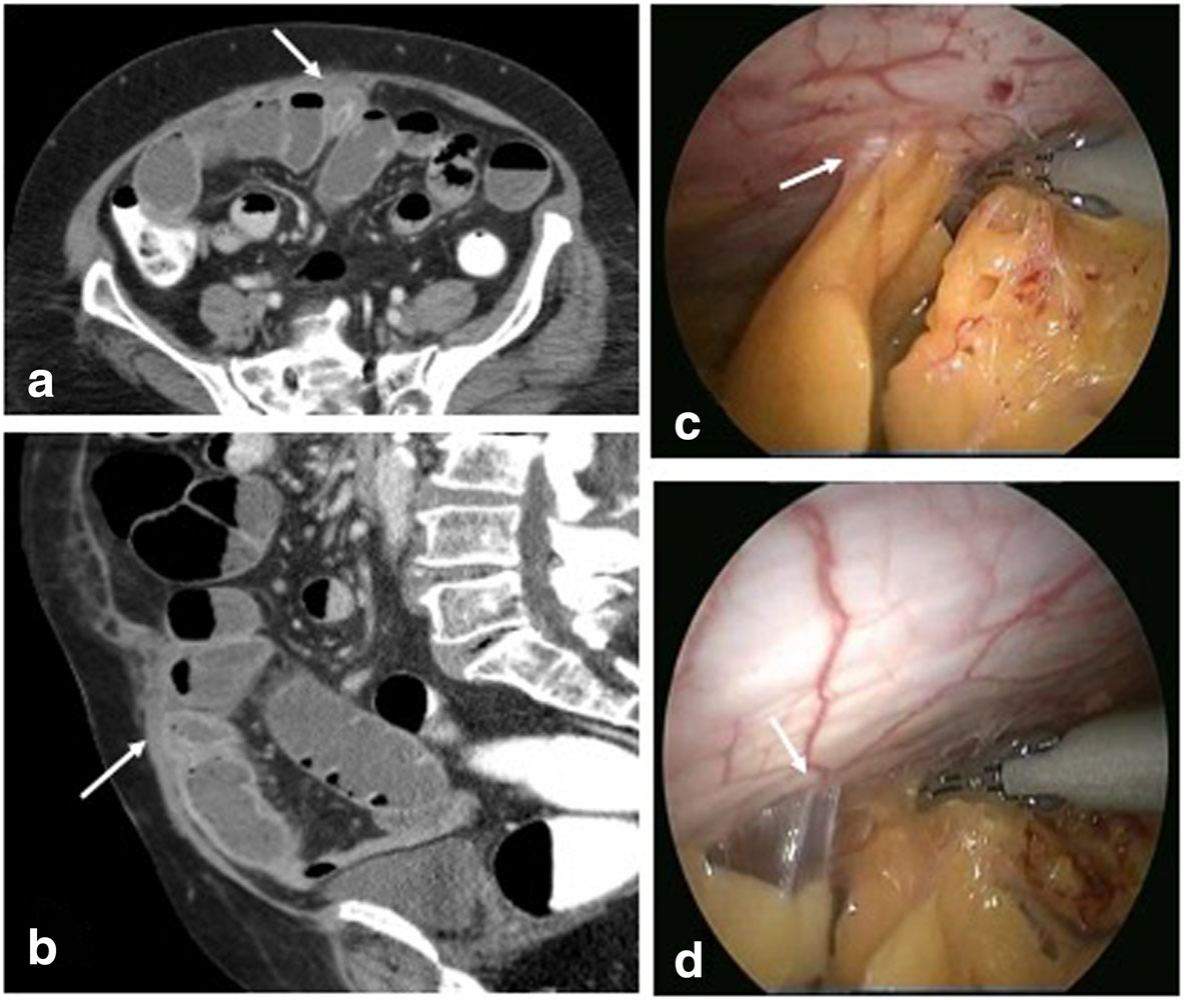
**a**, **b** CECT in axial and sagittal planes in a post ventral hernia repair shows bowel loops stuck to the anterior peritoneum (arrows). **c**, **d** Per operative image shows laparoscopic release of adhesions (arrow)

**Fig. 8:**
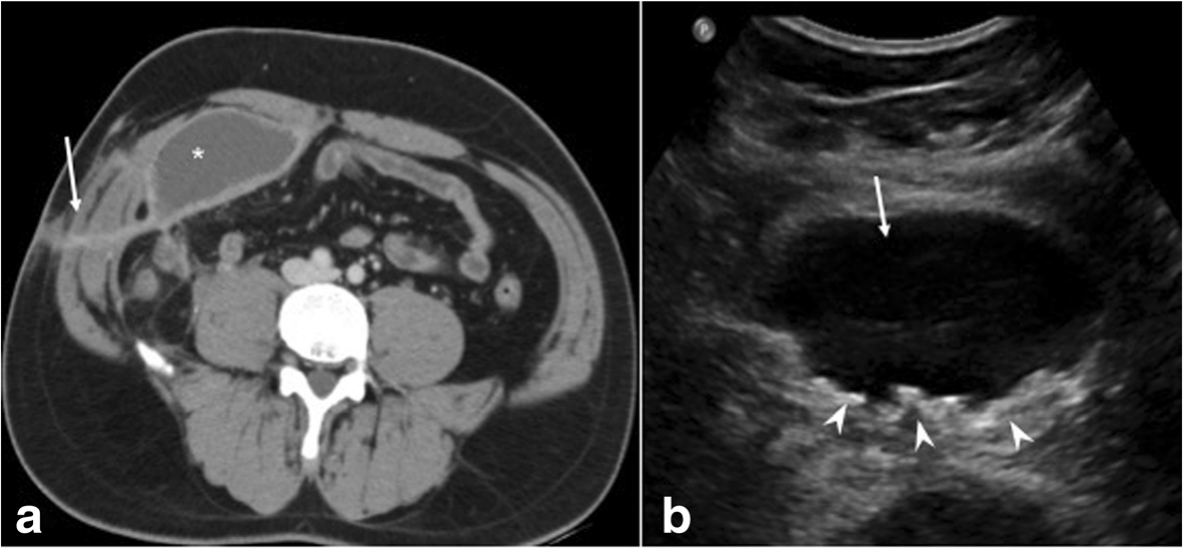
**a** CECT axial section in a stab injury related hernia post mesh repair shows infected collection (*) with sinus tracks (arrow). **b** USG of the same shows collapsed mesh (arrowheads) within the collection (arrow)

**Fig. 9:**
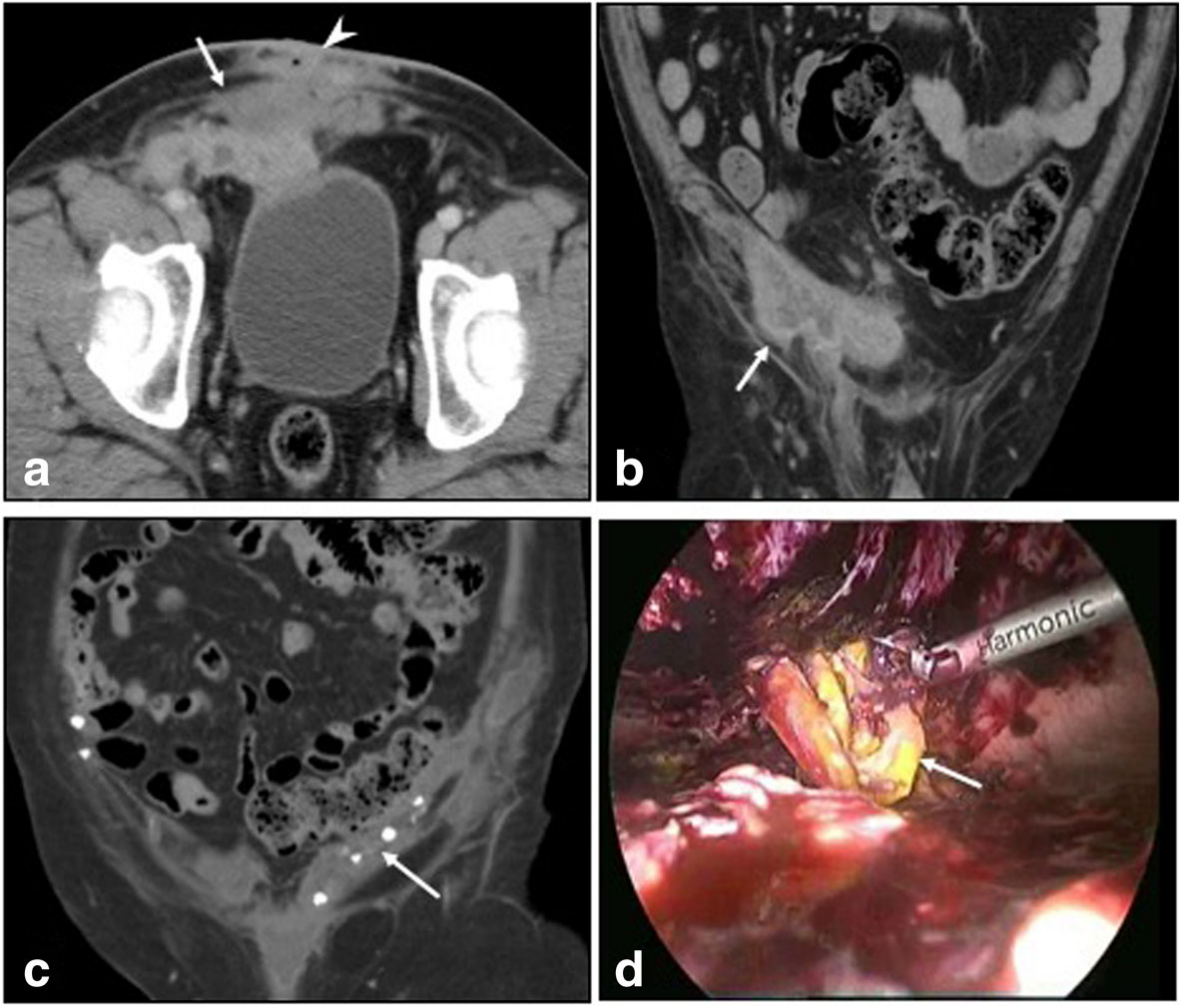
**a**, **b** CECT in axial and coronal sections show infected right inguinal hernia mesh repair with collections (arrows) and sinus tracks (arrowhead). Culture yielded *Mycobacterium chelonae*. **c** Coronal plain CT in another patient shows soft tissue thickening and collection along the mesh tackers (arrow). **d** Laparoscopic retrieval of infected mesh (arrow) which was covered with pus and granulation tissue

- Seroma: Seroma cannot be considered as a complication as it is a normal occurrence and is secondary to fluid collection comprising of blood and lymph due to dissection of tissue planes especially in laparoscopic approach. The size of the seroma is proportionate to the amount of dissection. Resolution occurs in 95% cases. Rana et al. [2] reported seroma incidence of 44% in their study where hernioplasty with onlay technique was used. Seroma is complicated if it persists more than 6 weeks, is symptomatic, or increasing in size [11]. It is important to distinguish between seroma and an infected collection as the latter requires drainage and specific antibiotic therapy. Imaging depicts seroma as a well/ill-defined collection within the dead space. Wall is thin or imperceptible with no or minimal enhancement. Usually, the collection is anterior to the mesh. Fat lobules can be seen within the seroma (Fig. 5). Infected seromas show thick, vascularized wall with exudative content and may be accompanied by signs and symptoms of inflammation. A diagnostic aspiration is helpful to identify the organism and provide antibiotic sensitivity profile.
- Hematoma: Hematomas usually resolve and are dependent on surgical technique. They are seen as ill-defined heteroechoic collections on USG or hyperdense on plain CT (Fig. 6). Rarely, they expand if there is active ooze which can be picked on contrast CT. Spermatic cord hematomas are common with inguinal hernia repairs and usually resolve [12].
- Adhesions: Adhesions can occur with any intraabdominal surgery and are the most common cause of small bowel obstruction in a postoperative abdomen. Use of composite mesh has shown to reduce the adhesions as well aiding in better meshoma formation [5]. On imaging, clues to adhesion-related obstruction include clumping of loops, adhesion of bowel to anterior abdominal wall, visualization of fat containing band, and acute angulation of loops at the site of transition (Fig. 7).
- Infection: Incidence of infection in post hernia repair is highly variable (0–36%) and is dependent on many factors. Common causative organisms include *Staphylococcus* (esp Methicillin resistant), *Streptococcus*, *Escherichia coli*, and anaerobic bacteria. Unique organism associated with implant infection include nontuberculous Mycobacterium which is seen in developing countries. Repair of complex hernias, hernias containing bowel and requiring bowel anastomosis, parastomal hernias, longer surgical time, equipment sterility, and impeded patient immune response have higher risk of infection. Microporous and multifilament mesh have higher predisposal to infection [5]. Mesh infection is generally managed by systemic antibiotics, drainage in case of abscess, and removal of mesh if deep infection is suspected. Removal of mesh is followed by resuturing/placement of biodegradable mesh which incite less tissue reaction. Infection can predispose to sinus formation, mesh migration, and erosion into viscera. Imaging helps in delineating the extent of infection and associated visceral involvement (Figs. 8 and 9).

Nontubercular Mycobacterium are resistant rapidly growing mycobacterium including common species such as *Mycobacterium chelonae*, *Mycobacterium goodie*, *Mycobacterium fortuitum*, and *Mycobacterium abscesses*. They have predilection for implants and are reported with hernia mesh, breast implants, and orthopedic prosthesis. Contamination occurs through inadequate high grade sterilization of instruments, rinsing with tap water and using partially dried instruments. These organisms have affinity for dermis and subcutaneous tissue and typically present at 3–4 weeks’ post-surgery appearing as an erythematous nodule followed by sinus formation [13].

**Fig. 10:**
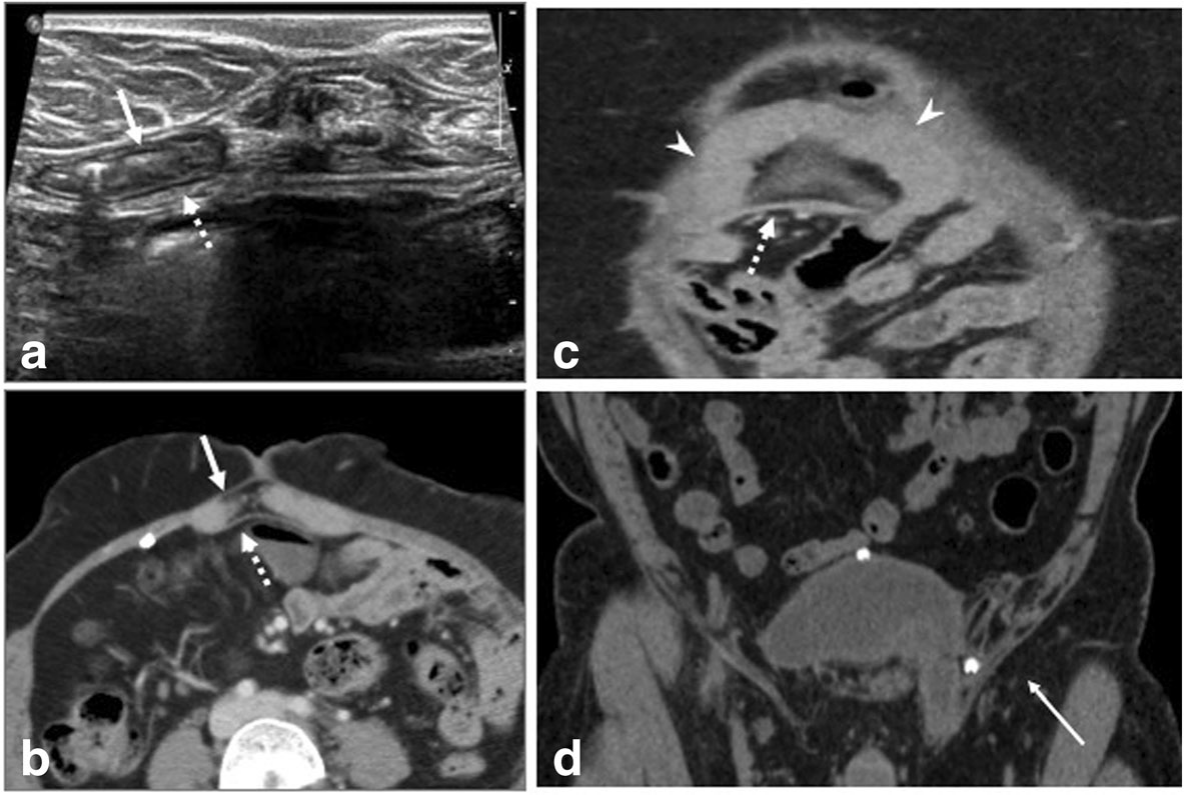
**a** USG shows recurrent herniation of the small bowel between the mesh (dashed arrow) and the parietal wall (white arrow). **b**, **c** CECT axial and coronal planes confirm the same with a loop of bowel (arrowheads in **c**) herniating between the inferior margin of the mesh (dashed arrow) and parietal wall (solid arrow). **d** Recurrent hernia containing urinary bladder (arrow) in a previously repaired inguinal hernia in a different patient

**Fig. 11:**
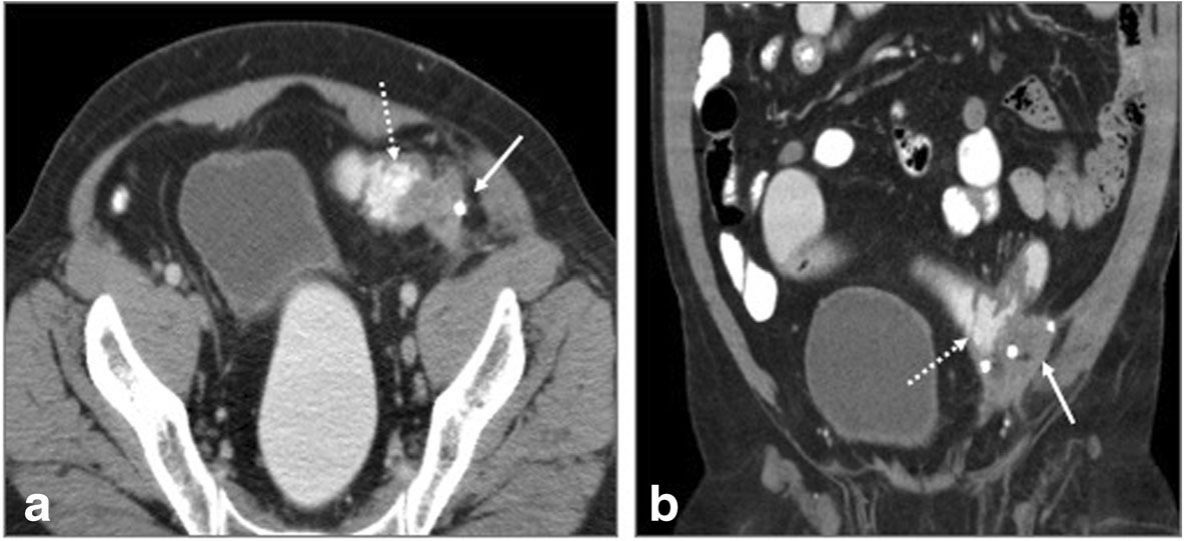
Post left inguinal hernia repair. **a**, **b** CECT axial and coronal sections show migrated mesh (arrow) breaching the sigmoid colon (dashed arrow). Per operatively, contained perforation of the colon was seen with adhered mesh

**Fig. 12:**
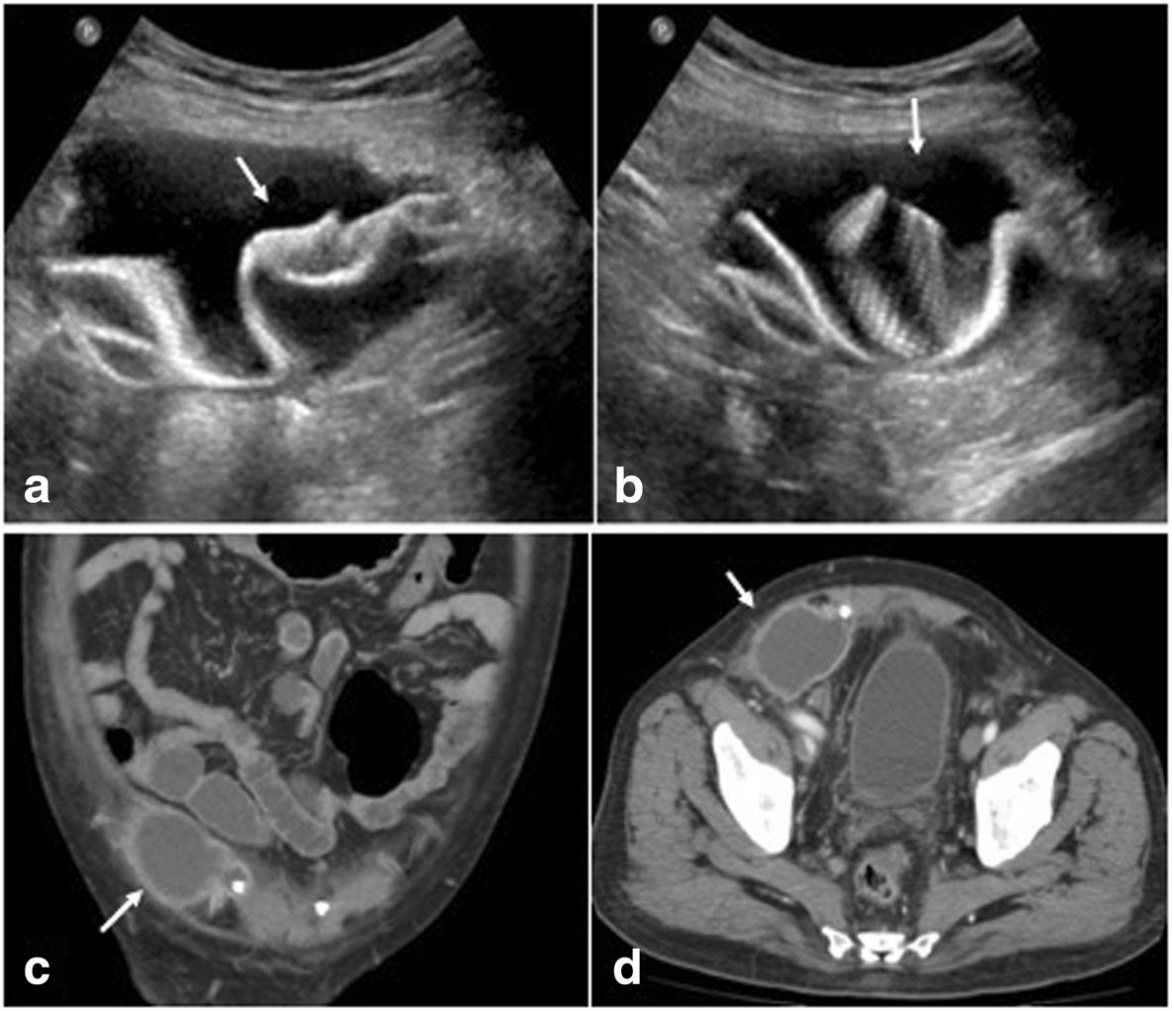
Post right inguinal hernia repair. **a**, **b** USG shows migrated crumpled mesh within the collection (arrow). **c**, **d** CECT of the same patient shows only the collection (arrow) demonstrating the added utility of USG in mesh localization

**Fig. 13:**
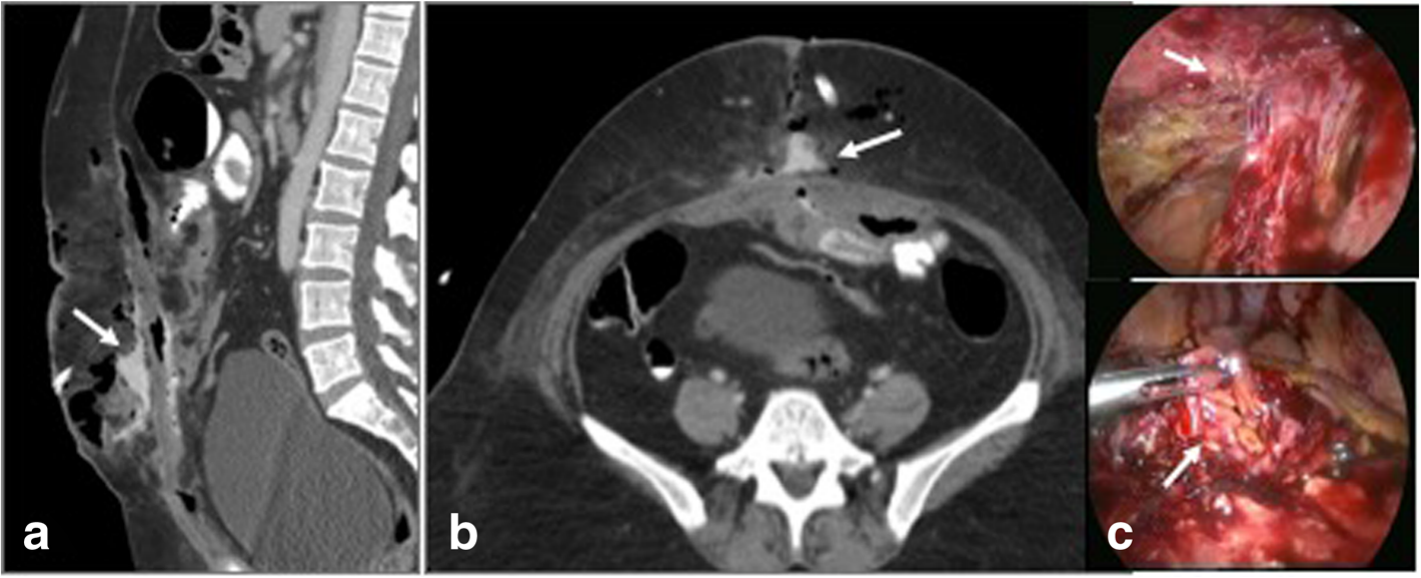
**a**, **b** CECT with oral positive contrast in a post ventral hernia repair shows small bowel fistulous communication showing extravasation of oral contrast through exterior (arrows). **c** Per operative demonstration of small bowel perforation and extensive adhesions (arrows)

**Fig. 14:**
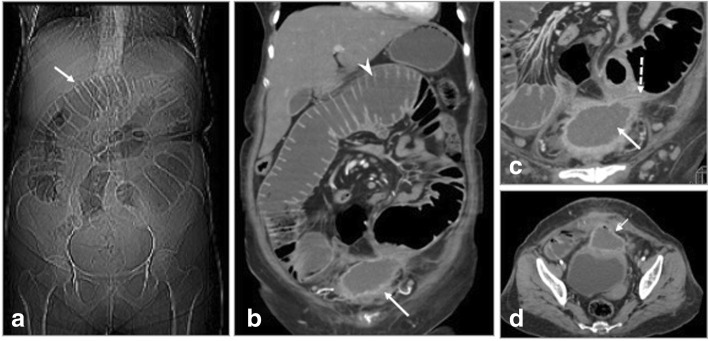
Multiple complications in a post ventral hernia repair. **a** CT scanogram shows signs of small bowel obstruction with severely dilated loops (arrow). **b**, **d** Coronal and axial CECT confirm the same (arrowhead) with collection suggesting mesh migration (arrow). **c** Magnified view of coronal section shows fistulization (dashed arrow) with small bowel

**Fig. 15:**
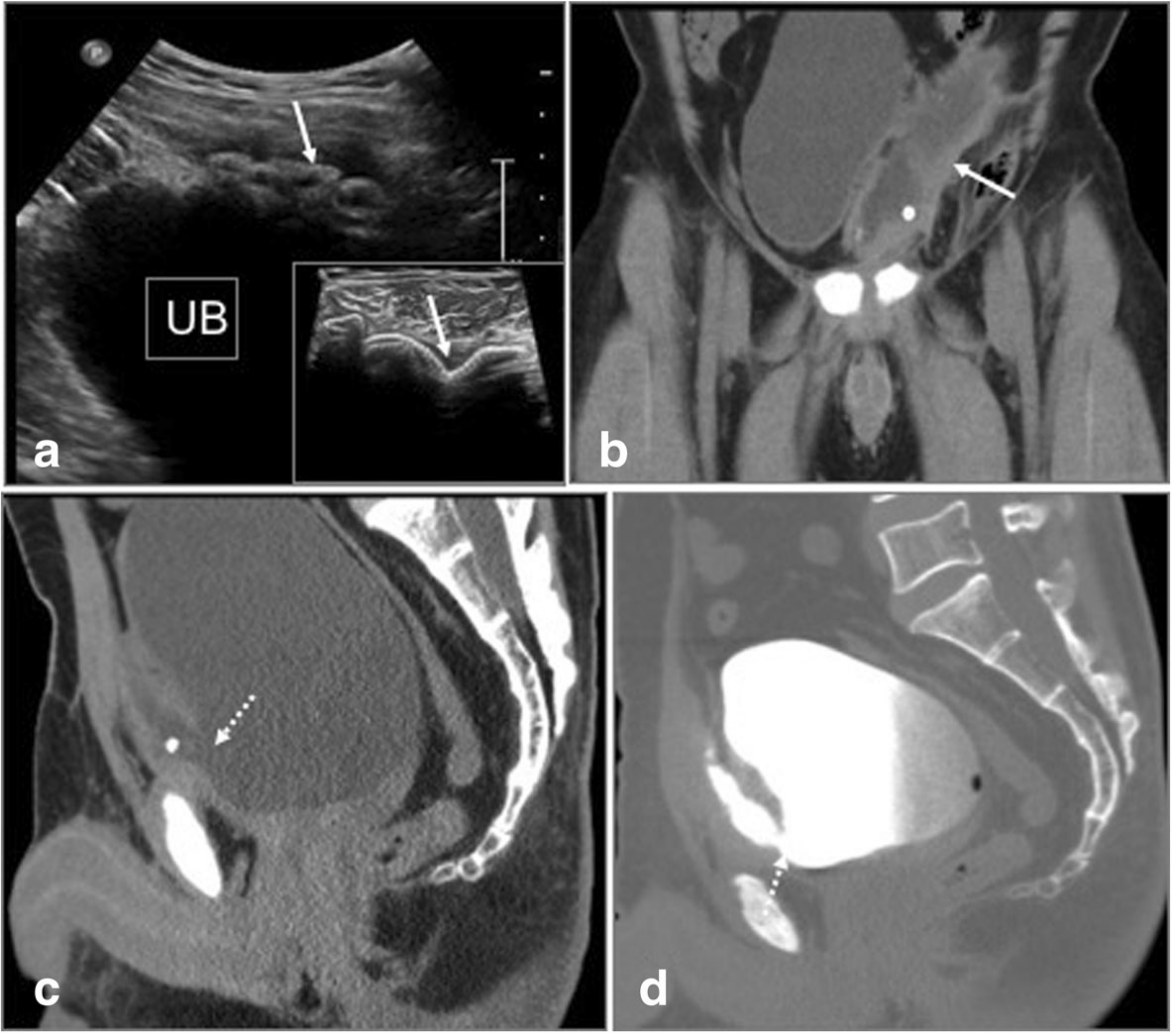
Post left inguinal hernia repair. **a** USG shows collection with migrated mesh (arrows) close to the urinary bladder (UB). **b**, **c** CT in coronal and sagittal planes show mesh eroding the anterior wall of urinary bladder (arrow) with fistulous communication (dashed arrow). **d** CT Cystogram confidently demonstrates the bladder communication (dashed arrow)

The non-healing sinus does not respond to routine antibiotics and is negative on aerobic/anaerobic cultures (Fig. 8). The clinical symptoms such as fever are absent. Imaging reveals sinus tracks with focal collections. No specific imaging feature of NTM infection is described. High clinical suspicion is needed based on above described presentation. Hospital outbreaks with atypical mycobacteria have been reported and are attributed to usage of common water reservoir harboring the pathogen [14]. Varying antibiotic combinations are used for treatment with most cases requiring mesh removal [15].

e. Recurrent hernia: Usually occurs within 2–3 years of surgery with an incidence ranging 0.3–10%. The frequency depends on surgical technique, obesity, post op complications, and relatively more with use of mesh with less tensile strength and mechanical stability [5]. Recurrence is common with repairs without mesh and open methods. Hernia recurrence invariably occurs at the margin of the mesh if used—at the “mesh—tissue” interface [16] (Fig. 10). Clinical diagnosis may be limited by overlying fibrosis and imaging is required in such cases. On sonography, the mesh margins should be evaluated utilizing Valsalva technique and without too much probe pressure to rule out reducible hernias.
f. Mesh migration: The exact incidence of mesh migration is unknown as reports are usually based on single case complications [17–21]. It generally occurs years later. The probable causes of mesh migration are divided into primary and secondary [22]. Primary is a mechanical migration where an inadequately secured mesh moves along planes of least resistance and secondary is through transanatomical planes as a result of inflammation, infection, and foreign body reaction. Mesh migration to urinary bladder, bowel, and scrotum has been reported with inguinal hernia and incisional hernia repairs. Migration into bowel can cause complications such as infection, obstruction, or fistula formation (Fig. 11). Collapsed mesh is best seen on USG as crumpled echogenic structure at an unexpected site or within a collection (Fig. 12). Tackers which are radiodense can aid in mesh localization on CT. Mesh migration, adhesion, and focal thickening of visceral wall suggest underlying erosion. Imaging is vital for management in providing details about viscera involved, extent of involvement and whether the migrated mesh can be removed endoscopically or require open removal.
g. Fistulization with adjacent viscera: It is a rare complication and is secondary to migration of mesh and erosion of adjacent viscera or due to deep infection resulting in extension to nearby organs or secondary to inadvertent inclusion of bowel/bladder in the sutures. Fistulization with small bowel, sigmoid colon, and urinary bladder has been reported in literature [18, 20, 21, 23] with both polypropelene and composite dual mesh (Figs. 13 and 14). Combined multimodality imaging approach (USG, CT) is preferred for diagnosis. Endoscopy can be used for diagnosis and retrieval of mesh in cases with colon involvement. CT protocol has to be modified according to clinical suspicion and use of positive contrast as in cystogram/oral route helps in fistula characterization (Fig. 15).
h. Miscellaneous

Direct organ injury: urinary bladder injuries are reported with laparoscopic inguinal repairs [12]. Bowel perforation is very rare and can lead to fistula, sepsis, and has increased mortality.

Infertility: injury to the vas deferens and testis at the time of surgery is ~ 0.3% for adults, 0.8–2.0% for children, and 0.5% of primary hernia repairs respectively [24]. Recurrent hernia repairs have higher rates of infertility. These are long-term complication and imaging is not used for diagnosis.

**Fig. 16:**
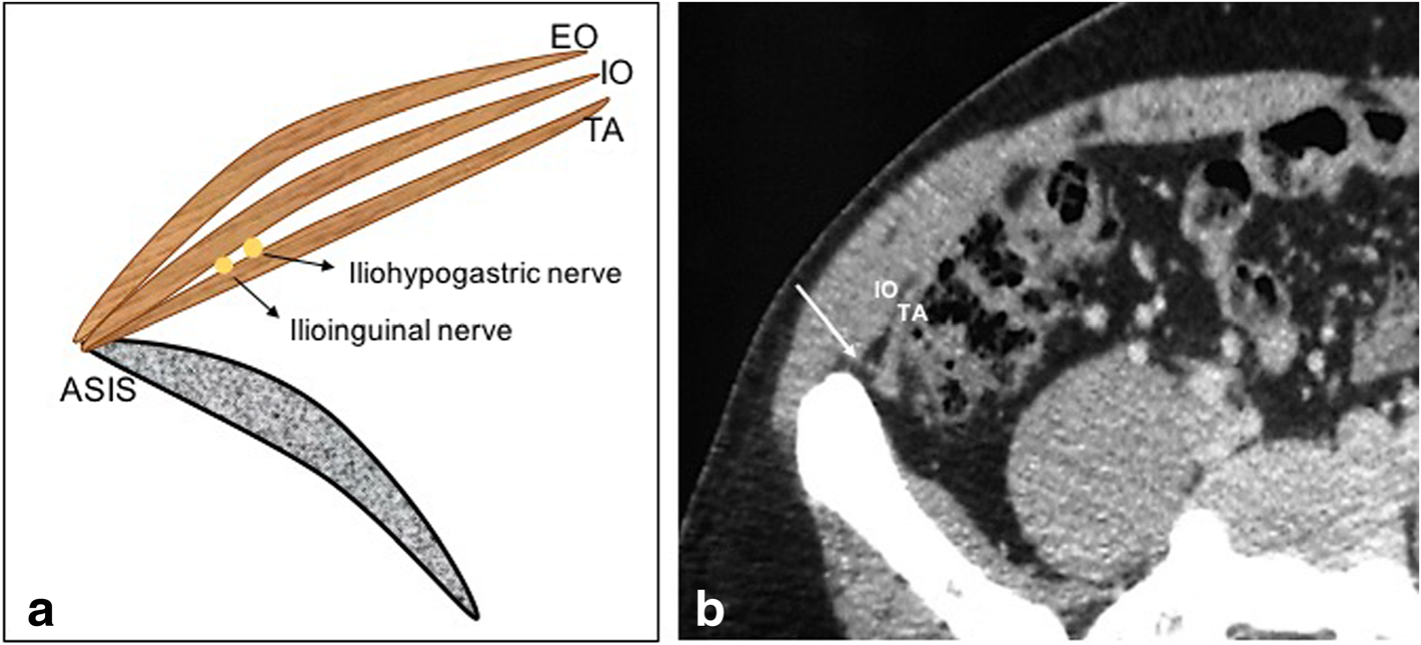
**a** Illustration of ilioinguinal and iliohypogastric nerve. *EO* external oblique, *IO* internal oblique, *TA* transverse abdominis, *ASIS* anterior superior iliac spine. **b** Axial CT at level of anterior superior iliac spine shows the nerves (arrow) between the transverse abdominis (TA) and internal oblique (IO) muscles

Post inguinal hernia surgery chronic pain: chronic groin pain is seen in up to 13–20% of individuals undergoing inguinal hernia repair [25]. Pain can be secondary to nerve damage intraoperatively or damage postoperatively due to neuroma formation, irritation by mesh margins, or by fibrotic reaction [25]. Use of laparoscopic techniques and light-weighted mesh have shown to reduce the incidence of chronic pain. In general, non-usage of sutures or tackers have shown to produce less pain as it provides a tension free repair. The nerves that are implicated include ilioinguinal, iliohypogastric, genital, or femoral branch of genitofemoral nerve and rarely lateral femoral cutaneous nerve. Chronic pain is usually dragging in nature, can be debilitating, and impede quality of life. Various management options ranging from pain medications to surgical neurectomy are undertaken. Imaging plays a role in providing guided ilioinguinal and iliohypogastric nerve injections/ablations [26]. Both CT and USG are used for localization, though the latter is commonly used (Fig. 16).

## Conclusion

Imaging provides a roadmap of the type and extent of complication. It is imperative that radiologists have prior knowledge about the indication and type of surgical procedure and also decide on the adequate modality choice to provide a fruitful report that aids in management of the complication.
